# A Cross-Sectional Study of Depression, Anxiety, and Insomnia Symptoms in People in Quarantine During the COVID-19 Epidemic

**DOI:** 10.3389/ijph.2022.1604723

**Published:** 2022-07-20

**Authors:** Chun Lin, Xiaohong Fu

**Affiliations:** ^1^ Department of General Medicine and Geriatrics, Shenzhen Qianhai Shekou Free Trade Zone Hospital, Shenzhen, China; ^2^ Department of Pediatrics, Shenzhen Qianhai Shekou Free Trade Zone Hospital, Shenzhen, China

**Keywords:** anxiety, COVID-19, quarantine, depression, cross-sectional study, insomnia

## Abstract

**Objective:** To investigate the status and influential factors of depression, anxiety, and insomnia among people in quarantine during COVID-19.

**Methods:** Data was collected from August 2020 to November 2021 through an online survey of 1,360 people in a quarantined hotel. The Patient Health Questionnaire-9 (PHQ-9), Generalized Anxiety Disorder-7 (GAD-7), and Insomnia Severity Index (ISI) were used to assess different mental symptoms.

**Results:** 19.9% (*n* = 270), 17.6% (*n* = 239) and 7.1% (*n* = 97) of participants had depression, anxiety and insomnia. Married (OR = 0.641, 95% CI = 0.450–0.915) was a protective factor for depression. Chronic disease (OR = 2.579, 95% CI = 1.416–4.698) was a risk factor for insomnia. No psychiatric medication history was a protective factor for depression (OR = 0.227, 95% CI = 0.068–0.757) and insomnia (OR = 0.240, 95%CI = 0.078–0.736). Female, history of mental illness, low moods at check-in, and partial/cannot understand the quarantine policies were risk factors for anxiety, depression, and insomnia.

**Conclusion:** People in quarantine had problems with depression, anxiety, and insomnia. Female, low moods at check-in, and partial/cannot understand the quarantine policies had significant impacts. It is necessary to help quarantined people understand quarantine policies, reduce negative emotions and improve sleep quality.

## Introduction

COVID-19 is the infectious disease of the world’s pandemic. As of 16 December 2021, the virus spread rapidly, leading to 271,376,643 confirmed infections and 5,324,969 deaths worldwide [1]. Quarantine is one of the effective ways to control the spread of COVID-19. WHO issued guidelines for quarantine measures to strengthen epidemic prevention and control. Mandatory mass quarantines inevitably have a marked psychological impact on the public, with the media reporting the number of new cases every day, resulting in panic and psychological stress [2]. Past experiences of quarantine [3], from SARS to Ebola, have shown that it can take a toll on a person’s mental health, including separation from loved ones, losing freedom, enduring illness, and feeling bored are among the most common problems. Anxiety and mood disorders are also prevalent [4]. A US study [5] reported an increased incidence of first psychiatric diagnosis (HR = 1.6–2.1) in COVID-19 patients within 14–90 days of COVID-19 diagnosis. Studies [6] linked quarantine to negative emotions and increased psychological stress, PTSD, and depression. A meta-analysis [7] of COVID-19’s effects on mental health among 33,062 participants noted that depression prevalence reached 22.8%, anxiety prevalence reached 23.2%, and insomnia prevalence reached 38.9%. The lack of data is a global problem. Only one study [8] conducted in the Czech Republic used structured diagnostic interviews (MINI) to assess prevalence in repeated cross-sectional surveys before and during the pandemic (2017 and 2020). A two—to three-fold increase in point prevalence of anxiety and depression has been reported. The Chinese government instituted an unprecedented nationwide public health home quarantine measure to control the spread of the pandemic. Some unique features of China’s COVID-19 epidemic pattern and its management policies contribute to the public mental health crisis [9]. Many people returning or leaving China were forced to stay in quarantined hotels due to local quarantine policies in cities across China. COVID-19 patients and healthy citizens unaffected have suffered from stress and anxiety caused by these massive quarantine policies. Unfavourable news can cause mental panic and negative emotions in people who are isolated for a long time [10]. In terms of policy, recognizing the epidemic’s impact on public mental health and life satisfaction and illuminating issues related to public mental health is crucial in large-scale quarantine measures to prevent infection in countries with high levels of COVID-19 [6].

## Methods

### Subjects

The subjects were in a quarantined hotel in Shenzhen from August 2020 to November 2021, all of whom were inbound from Hong Kong. After finishing the first nucleic acid testing at the customs, they would stay 14 days in the quarantine hotel. Inclusion criteria: 1) Occupancy date from August 2020 to November 2021; 2) Good cognitive ability, good reading comprehension ability, and able to complete the answers normally; 3) Complete the questionnaire survey on the day of check-in; 4) Informed consent to the questionnaire survey and voluntary participation. Exclusion criteria: 1) infants and illiterate persons; 2) Unable to understand the questionnaire’s content or incapable of completing the questionnaire due to cognitive impairment.

### Measures

The questionnaire was divided into two parts. The first part collected sociodemographic data, including gender, age, education, marital status, whether quarantine alone, history of physical illness, history of mental illness, history of psychiatric medication, moods at check-in, and attitude towards quarantine policies. Another part of the study focused on standard scales to measure depression, anxiety, and insomnia symptoms, such as PHQ-9, GAD-7, and ISI. There were nine questions on the PHQ-9 scale, and each question was scored 0–3 points, a total of 28 points. 0 to 4 points were for normal. In this paper, subjects were divided into 0 to 4 points for the normal group and >4 points for the depression group. There were seven questions on the GAD-7 scale, and each question was scored 0–3 points out of 21 points. 0 to 4 points were for normal people. This paper divided subjects into 0 to 4 points for the normal group and >4 points for the anxiety group. There were seven questions on the ISI scale, each graded on a scale of 0–4 points out of 28. 0–7 points for normal and 8–14 points for subclinical insomnia. In this paper, subjects were divided into 0–14 points for the normal group and >14 points for the insomnia group. An invalid questionnaire would be marked if the response time was less than 1 min or if there were more than 20% incomplete responses.

### Statistical Analysis

The statistical analysis was performed using SPSS 20.0. Continuous variables were expressed as mean ± standard deviation. Continuous variables were expressed by mean ± standard deviation. Classified variable data were expressed by numerical value or percentage. To compare the count data between groups, we used the Chi-square test. Fisher’s exact test was adopted when the expected frequency of grouped variables was less than 5. The correlation of data was analyzed using Spearman correlation analysis. Multivariate analysis was performed by binary logistic regression. Odds ratios (OR) and 95% confidence intervals (95% CI) of independent variables were calculated. *p* < 0.05 was considered statistically significant.

## Results

### Characteristics of the Participants

A total of 1,360 valid questionnaires were collected for data analysis. 639 were female (47.0%), and 721 were male (53.0%). The average age was 41.82 ± 11.4 years old. 77.7% were quarantined alone. Most of them were well educated, married, had no history of chronic physical illness, mental illness, psychiatric medication history, good moods at check-in, and fully understood the quarantine policies, as shown in Table 1.

**TABLE 1:** Descriptive statistics outcomes of difference factors of depression, anxiety and insomnia symptoms among people in quarantine during COVID-19 epidemic study. China, 2020–2021.

Subject	N (%)/Mean ± SD
Gender	
Male	721 (53.0)
Female	639 (47.0)
Age	41.8 ± 11.4
Adults	1,335 (98.2)
Old people	25 (1.8)
Whether quarantine alone	
Yes	1,057 (77.7)
No	303 (22.3)
Education	
Poor educated	137 (10.1)
Well educated	1,223 (89.9)
Marital status	
Single	429 (31.5)
Married	931 (68.5)
History of physical illness	
Healthy	1,081 (79.5)
Chronic disease	279 (20.5)
History of mental illness	
Yes	77 (5.7)
No	1,283 (94.3)
History of psychiatric medication	
Yes	67 (4.9)
No	1,293 (95.1)
moods at check-in	
Great	840 (61.8)
General	439 (32.3)
Bad	47 (3.5)
Very bad	34 (2.5)
Attitude towards quarantine policies	
Fully understanding	1,192 (87.6)
Partial understanding	160 (11.8)
Cannot understanding	8 (0.6)
PHQ-9	2.8 ± 4.8
Normal	1,090 (80.1)
Depression	270 (19.9)
GAD-7	2.2 ± 4.1
Normal	1,121 (82.4)
Anxiety	239 (17.6)
ISI	4.7 ± 5.89
Normal	1,263 (92.9)
Insomnia	97 (7.1)

SD, standard deviation; PHQ-9, Patient Health Questionnaire-9; GAD-7, Generalized Anxiety Disorder-7; ISI, insomnia severity index.

### Status of Depression, Anxiety, and Insomnia Among People in Quarantine

The mean PHQ-9 score was 2.8 ± 4.8. 270 (19.9%) of them were screened for depression. The average score of GAD-7 was 2.2 ± 4.1, and 239 (17.6%) of them were screened for anxiety. The average ISI score was 4.7 ± 5.9. Ninety-seven of them (7.1%) with insomnia were screened out, as shown in Table 1.

### Difference Analysis of Depression, Anxiety, and Insomnia Among People in Quarantine

Age, education, and whether quarantine alone showed no significant difference in the risk of depression, anxiety, and insomnia among people in quarantine (*p* > 0.05). Gender, history of physical illness, history of mental illness, history of psychiatric medication, moods at check-in, and attitude towards quarantine policies had statistical significance in the risk of depression, anxiety, and insomnia (*p* < 0.05). Marital status had no difference in the risk of anxiety and insomnia (*p* > 0.05) but had statistical significance in the risk of depression (*p* < 0.05). See Table 2.

**TABLE 2:** Chi-square test or Fisher’s exact test of difference factors of depression, anxiety and insomnia symptoms among people in quarantine during COVID-19 epidemic study. China, 2020–2021.

Subject	PHQ-9			GAD-7			ISI		
	N (%)	*X* ^2^/Fisher’s exact test	*p*	N (%)	*X* ^2^/Fisher’s exact test	*p*	N (%)	*X* ^2^/Fisher’s exact test	*p*
Gender		7.525	0.006		11.452	0.001		4.843	0.028
Male	123 (45.6)			103 (43.1)			41 (42.3)		
Female	147 (54.4)			136 (56.9)			56 (57.7)		
Age		—	0.802		—	1.000		—	0.697
18–64	266 (98.5)			235 (98.3)			95 (97.9)		
>64	4 (1.5)			4 (1.7)			2 (2.1)		
Whether quarantine alone		0.036	0.850		1.762	0.184		2.617	0.106
Yes	211 (78.1)			178 (74.5)			69 (71.1)		
No	59 (21.9)			61 (25.5)			28 (28.9)		
Education		0.073	0.787		0.530	0.467		0.609	0.435
Poor educated	26 (9.6)			21 (8.8)			12 (12.4)		
Well educated	244 (90.4)			218 (91.2)			85 (87.6)		
Marital status		9.286	0.002		1.742	0.187		3.629	0.057
Single	106 (39.3)			84 (35.1)			39 (40.2)		
Married	164 (60.7)			155 (64.9)			58 (59.8)		
History of physical illness		58.955	0.000		65.785	0.000		120.668	0.000
Healthy	169 (62.6)			144 (60.3)			35 (36.1)		
Chronic disease	101 (37.4)			95 (39.7)			62 (63.9)		
History of mental illness		205.307	0.000		232.569	0.000		449.553	0.000
Yes	64 (23.7)			63 (26.4)			52 (53.6)		
No	206 (76.3)			176 (73.6)			45 (46.4)		
History of psychiatric medication		188.403	0.000		193.239	0.000		—	0.000
Yes	57 (21.1)			54 (22.6)			47 (48.5)		
No	213 (78.9)			185 (77.4)			50 (51.5)		
Moods at check-in		339.736	0.000		297.355	0.000		141.739	0.000
Great	35 (13.0)			30 (12.6)			5 (5.2)		
Low	235 (87.0)			209 (87.4)			92 (94.8)		
Attitude towards quarantine policies		122.838	0.000		124.235	0.000		133.011	0.000
Fully understanding	183 (67.8)			158 (66.1)			49 (50.5)		
Partial/Cannot understanding	87 (32.2)			81 (33.9)			48 (49.5)		

PHQ-9, Patient Health Questionnaire-9; GAD-7, generalized anxiety disorder-7; ISI, insomnia severity index.

### Correlation Between PHQ-9, GAD-7, and ISI Scores Among People in Quarantine

Spearman correlation analysis showed that the PHQ-9 score was positively correlated with the GAD-7 score (r = 0.705, *p* < 0.0001). PHQ-9 score was positively correlated with the ISI score (r = 0.528, *p* < 0.0001). GAD-7 score was positively correlated with ISI score (r = 0.533, *p* < 0.0001).

### Influential Factors of Depression, Anxiety, and Insomnia Among People in Quarantine

Multivariate logistic regression analysis showed that being married (OR = 0.614, 95% CI = 0.450–0.915) was a protective factor for depression. Chronic disease (OR = 2.579, 95% CI = 1.416–4.698) was a risk factor for insomnia. No psychiatric medication history was a protective factor for depression (OR = 0.227, 95% CI = 0.068–0.757) and insomnia (OR = 0.240, 95% CI = 0.078–0.736). Female, history of mental illness, low moods at check-in, and partial/cannot understand the quarantine policies were risk factors for anxiety, depression, and insomnia. See Tables 3–5.

**TABLE 3:** Multivariate logistic regression analysis outcomes for influential factors of depression symptoms among people in quarantine during COVID-19 epidemic study. China, 2020–2021.

Subject	Variable	*p*	OR	95% CI
Sex	Female	0.024	1.484	1.052–2.093
Marital status	Married	0.014	0.641	0.450–0.915
History of mental illness	No	0.001	0.168	0.057–0.493
History of psychiatric medication	No	0.016	0.227	0.068–0.757
Moods at check-in	Low	0.000	15.266	10.092–23.094
Attitude towards quarantine policies	Partial/Cannot understanding	0.000	2.230	1.455–3.417

PHQ-9, Patient Health Questionnaire-9; OR, odds ratio; 95% CI:95% Confidence Interval.

**TABLE 4:** Multivariate logistic regression analysis outcomes for influential factors of anxiety symptoms among people in quarantine during COVID-19 epidemic study. China, 2020–2021.

Subject	Variable	*p*	OR	95% CI
Sex	Female	0.000	1.897	1.331–2.704
History of mental illness	No	0.000	0.095	0.032–0.281
Moods at check-in	Low	0.000	13.995	9.019–21.715
Attitude towards quarantine policies	Partial/Cannot understanding	0.000	2.304	1.491–3.561

GAD-7, Generalized Anxiety Disorder-7; OR, odds ratio; 95% CI:95% Confidence Interval.

**TABLE 5:** Multivariate logistic regression analysis outcomes for influential factors of insomnia symptoms among people in quarantine during COVID-19 epidemic study. China, 2020–2021.

Subject	Variable	*p*	OR	95% CI
Sex	Female	0.040	1.832	1.027–3.269
History of physical illness	Chronic disease	0.002	2.579	1.416–4.698
History of mental illness	No	0.000	0.109	0.040–0.298
History of psychiatric medication	No	0.012	0.240	0.078–0.736
Moods at check-in	Low	0.000	18.970	7.044–51.086
Attitude towards quarantine policies	Partial/Cannot understanding	0.001	2.950	1.603–5.430

ISI, insomnia Severity index; OR, odds ratio; 95% CI:95% Confidence Interval.

## Discussion

There was no large-scale epidemiological data to show the central psychological problems of the current epidemic and the main psychological problems in different populations. While the neurological effects of COVID-19 are still unknown, adverse effects of COVID-19 on physical and mental health are well documented [6]. United Nations Secretary-General Antonio Guterres released a policy brief on COVID-19 and mental health [11]. COVID-19 was not only affecting people’s physical health. Bereavement, unemployment, isolation, and movement restrictions lead to tremendous stress and mental health problems. A meta-analysis [12] of 134,061 patients revealed that COVID-19 quarantine was associated with anxiety, depression, and psychological stress. Quarantine and mental health are moderated differently by different groups. The country of origin had no significant effect on quarantine and mental health. Another meta-analysis [13] of 189,159 sample sizes revealed a prevalence of depression at 15.97%, anxiety at 15.15%, insomnia at 23.87%, PTSD at 21.94%, and psychological distress at 13.29%. Many studies [14–20] have shown that the outbreak of highly infectious diseases would bring about related mental symptoms, indicating that COVID-19 is an extremely stressful life event and a leading cause of physical and mental disorders.

### Depression, Anxiety, and Insomnia Were Positively Correlated

In COVID-19, the mental health study results varied significantly among countries [13]. A meta-analysis [21] indicates that depression, anxiety, and insomnia are prevalent among people in quarantine during the COVID-19 period. Those rates ranged from 0.9 to 48%, 0.7%–64%, and 0.9%–37.6%, respectively. Based on systematic reviews and meta-analyses [15] of coronaviruses (SARS, MERS, and SARS-CoV2), 14%–61% of those infected experience mental health complications, and 14.8%–76.9% experienced these problems after their illness. Our data showed a positive correlation between PHQ-9, GAD-7, and ISI scores. This positive correlation indicates that depression, anxiety, and insomnia were closely related and might have a high co-morbidity rate. Stress affects the internal state of balance between the body and mind, leading to physical and mental reactions such as tension, depression, anxiety, insomnia, or various physical problems [19]. Even if the pandemic is over, it to the general public health professionals and the influence on the vulnerable groups of mental health and well-being will continue for a long time. We want to be able to care for patients in a more personalized way [22].

### Multiple Regression Analysis on the Influential Factors of Depression, Anxiety, and Insomnia Among People in Quarantine

This study suggests that being married was a protective factor for depression. Single people increased the risk of depression by 35.9% compared with married people, consistent with existing research. Wang et al. [23] found that unmarried people suffered higher psychological stress during the pandemic. Tian et al. [24] showed that married people showed fewer psychological symptoms than unmarried people. Others reported more somatization, paranoid ideas, obsessiveness, depression, anxiety, phobias, and psychosis than married people.

Chronic disease increases the risk of insomnia, and psychiatric medication history contributes to depression and insomnia. Additionally, the risk of anxiety, depression, and insomnia increases with a mental illness history. Available evidence suggests that some of the measures taken to contain the pandemic may harm vulnerable populations, including those with mental or physical health problems and those with mental illness [6, 25, 26]. Due to the loss of access to mental health support and active activities, COVID-19 isolation can increase anxiety and depression in people coping with it.

Female, lack of understanding of quarantine policies (including partial and cannot understand at all) and low moods (including general, bad, and very bad moods) at check-in would significantly increase the risk of depression, anxiety, and insomnia. This study showed that females were 1.484, 1.897, and 1.832 times more than males to experience the risk of depression, anxiety, and insomnia, respectively. When previous trends are considered, mental distress is higher than expected, especially among women. Consistent with many existing studies [27–29]. In an earlier cross-sectional study of COVID-19 in Spain (*n* = 3,480), the female was a strong predictor of anxiety. A meta-analysis [7] also noted a higher prevalence of anxiety among women during the pandemic. According to a Greek study [30], women were more likely to have sleep problems, reflecting an existing gender difference in anxiety and insomnia symptoms.

China continued to implement the most stringent prevention and control measures globally [31], including nucleic acid testing, tracking, self-isolation, quarantine, and broader population measures, including travel bans, school closures, assembly restrictions, curfews, and total lockdown. Entry quarantine was an essential means of preventing imported cases of COVID-19. However, some people still did not fully understand and agree with the quarantine policies. This study showed a positive correlation between the attitude towards quarantine policies and the moods at check-in (r = 0.361, *p* < 0.0001). The psychological resistance to quarantine policies would be reflected in the emotion during quarantine, resulting in adverse effects. More obvious negative emotions were observed for people in quarantine as a particular group. The low moods at check-in reflected the negative coping emotion of people in the face of the epidemic and quarantine policies. The psychological stress level of people with negative coping emotions was higher [23]. Negative coping emotions might be related to psychological stress or mental illness such as anxiety and depression [32, 33]. Considering the positive correlation between the attitude towards quarantine policies and the moods at check-in, people would have more pronounced negative emotions under quarantine measures [34]. In the study [35] of psychological interventions for people in quarantine, members of the psychological support team mentioned the experience of communicating with people in an equal manner, explaining the importance of quarantine measures, and acknowledging their contribution to social security. On 26 January 2020, China’s National Health Commission released guidelines for emergency psychological crisis intervention during the COVID-19 epidemic [36]. The notice provisions, psychological crisis intervention should be part of the public health response, by the municipal city and provincial levels organize joint prevention and control mechanism, interventions should be distinguished according to the group intervention staff. With the implementation of crisis interventions in China to reduce the negative psychosocial impacts on public mental health, there are challenges to mobilizing an effective mental health care system [22], which should provide which type of intervention, which groups need help, and in what way. In addition to emergency psychological crisis intervention measures afterward, this study suggests how to allow the public to understand different policies during different pandemic periods, which may significantly reduce various emotional and spiritual problems in quarantine. This involves official media must be authoritative and credible, rather than simply creating information panic to attract traffic. There is a risk that fake news can spread faster than the virus itself and create uncertainty and fear. This should be regulated through constant interaction with the media and national regulations.

### Limitations

This study is a single-center study without considering the impact of the difference in living environments and medical conditions from other quarantine sites on mental health problems. The research results might be biased. In addition, due to the limitation of conditions, there was no comparative investigation of mental health before and after quarantine and psychological intervention research.

### Conclusion

People in quarantine had problems with depression, anxiety, and insomnia. Depression, anxiety, and insomnia are closely related and positively correlated. Being married was a protective factor for depression. Chronic disease was a risk factor for insomnia. No psychiatric medication history was a protective factor for depression and insomnia. Female, history of mental illness, low moods at check-in, and partial/cannot understand the quarantine policies were risk factors for anxiety, depression, and insomnia. Positive correlations were found between attitudes towards quarantine policies and moods at check-in. In the face of public emergencies such as COVID-19, the psychological status of quarantined people will constantly change with the epidemic’s progress. Quarantine is part of our public health, but side effects must be weighed against alternatives like every medical intervention [2]. For example, voluntary isolation may be associated with good compliance and less psychological impact, especially when clearly explained and advertised as altruism. Therefore, timely monitoring and intervention of psychological status should be carried out during quarantine, but how to make the public understand quarantine policies during different periods deserve further studies.
